# The neuromuscular factor in idiopathic scoliosis; Retrospective longitudinal study of a group of 308 adolescents treated with neuromuscular rebalancing

**DOI:** 10.1186/1748-7161-8-S1-O4

**Published:** 2013-06-03

**Authors:** A Fimiani

**Affiliations:** 1 Ischia, Italy

## Background

One of the etiologic agents of Idiopathic Scoliosis (IS) is the sensorineural and/or neuromuscular factor. J.P. Roll demonstrated that: 1) the muscle spindle is the postural adjustment source, 2) the right balance between agonist and antagonist muscles define the posture, 3) the feet are the gravity reference to the central nervous structures and 4) the sub-occipital muscles connect the head muscles with myofascial body lines. Through the theory of the myofascial lines, T. Myers demonstrated the functional continuity of the muscular system in its entirety, thus justifying the huge sensitivity of the Postural System, and the opportunity to act on the myofascial lines by several receptor inputs. The neuromuscular imbalance is a factor favoring the onset, and conditioning the progress, of the IS. The neuromuscular error manages the irregularity of scoliosis below a 25° Cobb angle, accordingly, neuromuscular rebalancing involves a spine realignment to the gravity axis, making an improvement of the curves by braking the worsening push, and producing a clear improvement in aesthetic damages.

## Aim

To present the results of neuromuscular rebalancing on adolescents with IS.

## Methods

Retrospective longitudinal study. In the group of 308 adolescents (193 female and 115 male, age between 7 and 14 years) 182 completed the therapeutic protocol of 24 months neuromuscular rebalancing. At the starting visit, 50 had an aligned spine, 45 a scoliotic attitude, and 87 an IS (33 thoraco-lumbar, 30 double curve, 24 lumbar). All patients were: 1) checked by x-rays, 2) initially Risser zero and 3) carried out only the Neuromuscular Rebalancing according to B. Bricot.

## Results

Considering the variations equal to, or greater than, 5° Cobb, among the 24 patients with lumbar scoliosis, only one of them (4.17%) had a curve worsening; among the 33 with a thoracolumbar 4 (12.2%); and among the 30 with a double curve 11 (36.6%). The aesthetic damage improves in all patients.

## Conclusions

The multi-sensory cocktail managing the automatic balance of the Postural System is certainly one of the idiopathic scoliosis etiological factors, probably the predisposing factor to the onset, and conditioning the disease evolution. The double curve scoliosis remains always the most evolutionary. The aesthetic damage in IS is mainly dependent on the Neuromuscular System failure.
